# Exploring health literacy development through co-design: understanding the expectations for health literacy mediators

**DOI:** 10.1093/heapro/daaf003

**Published:** 2025-02-28

**Authors:** Madeline Spencer, Vaughan Cruickshank, Nenagh Kemp, Rosie Nash

**Affiliations:** School of Medicine, College of Health and Medicine, University of Tasmania, 17 Liverpool Street, Hobart, Tasmania 7001, Australia; School of Education, College of Arts, Law and Education, University of Tasmania, Newnham Drive, Newnham, Tasmania 7001, Australia; School of Psychological Sciences, College of Health and Medicine, University of Tasmania, Grosvenor Street, Sandy Bay, Tasmania 7001, Australia; School of Medicine, College of Health and Medicine, University of Tasmania, 17 Liverpool Street, Hobart, Tasmania 7001, Australia

**Keywords:** health literacy, co-design, health inequity, social determinants of health, health literacy mediator

## Abstract

Health promotion efforts that enhance health literacy among individuals, organizations, and communities are gaining attention globally. Additionally, co-designed and community-led health promotion interventions have gained recognition as an effective strategy for addressing health issues through more effective and sustainable health outcomes. This qualitative study conducted in Tasmania, Australia, aimed to co-design the emerging Health Literacy Mediator (HLM) and to assess the support, expectations, and need for such a role. Following an Ophelia approach, data for this research were collected via focus group discussions within an online workshop, enabling diverse perspectives to emerge and be analysed via thematic analysis. Discussions around how the role could impact the individuals and families presented in the short case scenarios (vignettes) produced four strong expectations: that they are solution-focused, that they have a duty to facilitate change, that the role is based in community, and that the role targets those in the community who need them most to ensure the greatest impact. Participants also shared other recommendations and supporting ideas for the role. The potential role of HLM holds substantial promise in addressing health inequities for all individuals, regardless of background or socioeconomic status, by optimizing time management, simplifying navigation, ensuring the right care, and building community trust. By creating connections and advocating for individuals, HLMs could effectively break down barriers to healthcare access. This proactive approach not only addresses immediate health concerns but could also lay the groundwork for sustained improvements in public health outcomes in the future.

**Contribution to Health Promotion**

- Health literacy enhances individuals’ ability to access, understand, and use health information, which empowers them to engage in health-promoting behaviours and participate in collective health promotion actions.
- A Health Literacy Mediator (HLM) holds substantial promise in addressing health inequities by building health literacy assets (optimizing time management, simplifying navigation, ensuring the right care, and building community trust).
- Our methodology of co-design allows for the opportunity of community-led health promotion by engaging community members, researchers, policy-makers, and other stakeholders to discuss their expectations throughout the development process of the HLM role.

## INTRODUCTION

### Understanding health inequities

Health inequities are systematic differences in health status among individual population groups, influenced by factors such as the social determinants of health (SDH) (World Health Organization [WHO] 2018, 2024). These disparities stem from historical and contemporary inequities shaped by societal structures and unequal distribution of power and resources (National Academies of Sciences, Engineering, and Medicine 2017). They negatively impact some individuals and societies, leading to poor health outcomes, economic costs, and social disparities (Arcaya et al. 2015). Health inequities are closely linked to non-communicable diseases (NCDs) (WHO 2023). Globally, NCDs are a significant health concern, responsible for over 40 million deaths annually, with cardiovascular disease being the leading cause, followed by cancers, respiratory diseases, and diabetes (Andrade et al. 2023). These diseases are preventable and often linked to modifiable lifestyle factors such as smoking, alcohol use, physical inactivity, and unhealthy diets (WHO 2023). Addressing NCDs and health inequities involves coordinated national and international action, focusing on modifiable risk factors, improving access to high-quality chronic care management, and understanding root causes (such as health literacy [HL]) to then inform policies that reduce these disparities and meet the needs of the population, and especially vulnerable groups within it (Andrade et al. 2023; Wilkins et al. 2023).

### Health literacy as a key to equity

HL plays a crucial role in addressing both health inequity and NCDs by empowering individuals to understand health information, make informed decisions, and engage in self-management. Efforts to improve HL, both traditional and digital, are essential for promoting better health outcomes and reducing the burden of NCDs globally (Fitzpatrick 2023). The current state of HL within the Australian population reveals both strengths and challenges (Australian Bureau of Statistics 2019). People with greater HL challenges often experience adverse health outcomes, increased hospitalizations, and poorer health behaviours than those with fewer such challenges (Coughlin et al. 2020, Stormacq et al. 2020, Schillinger 2021). Improving the HL environment through effective communication strategies, embedding HL into policies, and ensuring accessible information can enhance health outcomes and quality of care (Sharkiya 2023). Knowing more about an individual’s and a community’s HL provides an important foundation when creating strategies to strengthen or maintain HL assets. HL assets can refer to the skills, knowledge, and resources individuals and communities possess to access, understand, appraise, and use health information effectively; these assets are vital as they empower people to make informed decisions, navigate healthcare systems, and engage in health-promoting behaviours (Bröder et al. 2019). Whether an individual has the required HL assets required to manage their health, may reflect on their HL strengths and challenges. This paper will investigate a new role focused on creating HL learning opportunities within a community, evaluating the support, expectations, and requirements for this role to inform future implementation. Enhancing HL assets can lead to better health outcomes, bolster health promotion initiatives, and improve overall well-being.

Health promotion and HL are distinct yet complementary concepts that together can contribute to improve overall health outcomes. Health promotion focuses on enabling individuals and communities to increase control over and improve their health through broad actions aimed at addressing social, environmental, and individual factors (WHO 2021). This includes implementing policies, providing education, and creating supportive environments that facilitate healthier choices (WHO 2021). In contrast, HL refers to an individual’s capacity to obtain, process, and understand basic health information needed to make appropriate health decisions (Gugglberger 2019). It encompasses people’s knowledge, motivation, and competences to access, understand, appraise, and apply health information effectively (Chahardah-Cherik et al. 2018). While these concepts differ in their scope and focus, they complement each other in several ways. HL serves as a foundation for effective health promotion, as individuals with stronger HL assets are better equipped to engage with and benefit from health promotion activities (Gugglberger 2019). Conversely, health promotion efforts often aim to improve HL as one of their outcomes, enhancing people’s health knowledge and skills through various educational initiatives. Both concepts share the ultimate goal of empowering individuals and communities to take control of their health and are critical for addressing health inequities and achieving broader health and development goals (WHO 2021). HL can be viewed as both an outcome of health promotion efforts and a tool that enables further health promotion (Gugglberger 2019). In essence, while health promotion provides broader strategies and actions to improve health, HL equips individuals and communities with the skills to effectively engage with these efforts and make informed health decisions. Together, they can create a more comprehensive approach to improving population health.

### The role of co-design in health promotion

Co-designed and community-led health promotion interventions have gained recognition as an effective strategy for addressing complex health issues while ensuring cultural appropriateness and local relevance. This collaborative approach involves engaging community members, researchers, policy-makers, and other stakeholders throughout the development and implementation of health initiatives (Jessup et al. 2018, Vargas et al. 2022). By embracing co-design, health promotion efforts can better address health inequities, enhance cultural competence, and lead to more effective and sustainable health outcomes (Ward et al. 2018, Duncan and Kolt 2019). Additionally, these approaches consider varying levels of HL within communities, making information and interventions accessible and understandable to all (Jessup et al. 2018, Elmer et al. 2022). International groups such as the WHO promote using a co-design process to co-design HL solutions (WHO 2022). An example of this is Optimizing Health Literacy and Access (Ophelia) process, which aims to improve HL and equitable access to healthcare by implementing locally tailored, evidence-informed solutions in collaboration with communities and stakeholders (Batterham et al. 2014). This approach begins by assessing the HL requirements of the intended population using the Health Literacy Questionnaire (HLQ) (Batterham et al. 2014). The HLQ was created to capture the multi-dimensional nature of HL (Osborne et al. 2013). The Ophelia approach then utilizes data-driven vignettes (case studies derived from HLQ data) to illustrate and convey the HL needs of the target population. This approach has been successful in the co-design of ideas to enhance the HL assets, responsiveness, and outcomes in numerous settings (Melwani et al. 2023, Tan et al. 2023).

Given the international literature above highlights that communities are experiencing significant HL challenges health promotion efforts must be cognisant of HL in their design, the concept of a Health Literacy Mediator (HLM) has been inspired by the Marmot Review: Fair Society, Healthy Lives (Marmot and Bell 2012), which highlighted the success of local health trainers and community champions in empowering individuals to manage their health. Similar roles have already been explored in Eastern Europe, for example, health mediators have been effective in bridging healthcare access for the Roma communities (Roma Health Mediators Project), and in Hungary, the integration of health mediators as part of multidisciplinary teams has shown success in addressing complex health needs and building trust (Kósa et al. 2020). Furthermore, various health-support roles such as health navigators, health connectors, health coaches, and health advocates have emerged internationally, reflecting a growing focus on improving, adapting, and developing HL practices. For example, health navigators, also known as patient navigators or care coordinators, help individuals overcome barriers to care by connecting them with healthcare providers and community resources (Budde et al. 2022). Health coaches use evidence-based strategies and techniques, such as motivational interviewing, to support patients in achieving health goals and integrating healthy habits into their lives (Perlman and Abu Dabrh 2020, Jordan 2021). Health advocates provide case management-like support, helping individuals ask questions and navigate the complexities of healthcare systems (Boroumand et al. 2020). Health connectors focus on addressing inequities by building social support networks for individuals and carers (Dolovich et al. 2020). Building on these foundational ideas, the current research team has expanded and formalized the new conception of the HLM role to address the specific needs and context of the Tasmanian community. Spencer et al. (2021), defined an HLM as ‘a person or group of people dedicated to providing learning experiences and opportunities to enable individuals and communities to overcome inequities perpetuated by their social determinants and increase their HL assets to improve their health outcomes’. This definition of the role indicates a holistic approach to supporting an individual’s healthcare journey, wherein there is a significant focus upon building autonomous capacity for all individuals, addressing local health inequities, and targeting those disadvantaged by their SDH (Spencer et al. 2021). The HLM role aims to improve comprehensive HL, beyond just healthcare access, and actively engage in health promotion with individuals, organizations, and policy-makers in the local community.

Community expectations of healthcare typically encompass accessible, affordable, and high-quality services (Lakin and Kane 2022). Additionally, communities desire healthcare systems that are culturally sensitive, equitable, and inclusive, ensuring that all individuals, regardless of background or socioeconomic status, receive adequate care (Elbrink et al. 2023). There is also an expectation for healthcare to be proactive in promoting health and preventing diseases through education and community-based interventions (WHO 2021). Investigating the role of HLMs is important due to their potential to impact an individual’s or community’s health outcomes positively. HLMs could bridge the gap between healthcare providers and patients, ensuring that individuals understand health information and can utilize and navigate the healthcare system effectively. This is particularly important for managing and preventing NCDs, which require ongoing patient engagement and self-management. By improving HL assets, HLMs could empower individuals to make informed health decisions, adhere to treatment plans, and adopt healthier lifestyles. This empowerment may lead to better management of chronic conditions, reduced hospital readmissions, and overall improved health outcomes (Small et al. 2013, Stepanian et al. 2023). Moreover, HLMs could play a pivotal role in addressing health inequities by targeting interventions towards disadvantaged populations, thus ensuring that HL improvements are inclusive and equitable (Spencer et al. 2024c). This is why this study aims to co-design the emerging HLM role with various stakeholders working in health and health-related settings across diverse Tasmanian regions. This will be achieved by assessing the support, expectations, and need for such a role via online workshops.

## METHODS

A collaborative constructivist approach was employed in this research. This approach was selected to explore and co-construct meaning from the data through active collaboration amongst researchers and participants (Creswell and Creswell 2018). The project received ethics approval from the University of Tasmania Research Ethics Committee (Approval Number H0026170). All participants were required to read an information sheet and give electronic and verbal consent prior to admission to the interview, they were aware that whilst within the workshop they were not anonymous to each other, but any data gathered during the discussion would be de-identified.

### Participants and recruitment

The study setting for this research was Tasmania, Australia. HL levels vary across Australia, with Tasmania experiencing some of the lowest health and educational outcomes, as highlighted by *the Optimising Health Care for Tasmanians* Report, which underscore the state’s challenges in addressing preventable chronic diseases, socioeconomic disadvantage, and educational attainment (Elmer et al. 2022). Due to the online nature of the workshop participants could be any within the state and still partake. Public health professionals, healthcare providers, managers, and allied health professionals working in Tasmania’s health sector were recruited through purposive and snowball sampling methods (Liamputtong et al. 2016). Recruitment occurred via the Tasmanian Health Literacy Network, the Tasmanian Health Department, and the research team’s professional networks. These stakeholders were selected for their relevant knowledge, experience, and interest in the project. Initial contact was made through an email from the research team, which included a brief study description and a registration form for participation in the co-design workshops. Interested individuals were then sent detailed participant information sheets and consent forms. The research team also encouraged these stakeholders to disseminate the workshop details within their own professional networks to increase participation.

A total of 15 stakeholders participated and chose one of two identical workshops to attend (Workshop 1, *n* = 8, Workshop 2, *n* = 7). The stakeholders represented a range of different sectors including the Department of Health, the University of Tasmania, and not-for-profit organizations, as summarized in Table 1. Participants were from all around the state with nine from Southern Tasmania, four from Northern Tasmania, and two from the Northwest Coast. The majority of the participants who took part in the workshops were women (*n* = 14).

**Table 1.** Departments/organizations represented by participating stakeholders.

Department or organizations	Number of stakeholders *N* = 15
Department for Education, Children and Young People	1
Public Health Services, Department of Health	3
Tasmanian Health Service (THS)	2
University of Tasmania	4
Community not-for-profit agency	1
Health not-for-profit agency	4

### Data collection

Consistent with the Ophelia approach, the data for this phase of the research project were gathered from focus group discussions within online workshops. Gathering data through co-design workshops aligns with one of the steps within the Ophelia process, where stakeholders collaboratively design tailored HL interventions based on identified community needs (Batterham et al. 2014). Two online workshops were conducted in March 2023 on Microsoft Teams, a conferencing software (Microsoft 2023). They went for approximately 1 hour each, first starting with an introduction to the overall project and an overview of the HLQ survey results from previous phases of the research project (Spencer et al. 2024a,b,c). Following this, data-informed vignettes were shared with the group. These vignettes were created specifically for this study from a cluster analysis of the HLQ data (*n* = 255) and interview data (*n* = 14) representing the HL strengths and challenges of the target population (Spencer, et al. 2024a). The following questions were presented with the aim of generating discussion to identify local solutions that could respond to the needs of the individuals and families personified in the vignette(s). The questions were:

- Do you know anyone like this individual or this family in your community?
- What are the main barriers that this individual/family is facing?
- What can be done to help this individual/family?
- How might an HLM assist in these solutions?
- Should an HLM role be an extension of what already exists or a new role?
- Would improving HL assets from an earlier age impact these situations?

Participants were encouraged to use their microphones and the chat function to contribute to discussions and share ideas during the workshop. Both the workshops were digitally recorded, with consent obtained from all participants beforehand. MS conducted all the workshops, with RN serving as the co-facilitator. Throughout and at the conclusion of each workshop, both the facilitator and co-facilitator made observational notes on the discussions that had occurred.

### Data analysis

**Figure 1.**
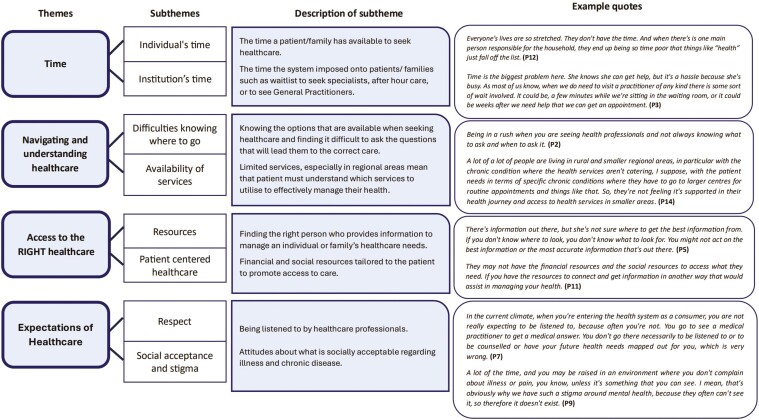
Co-design of barriers to healthcare that stakeholders identified from the vignettes during ideas generation workshops

**Figure 2.**
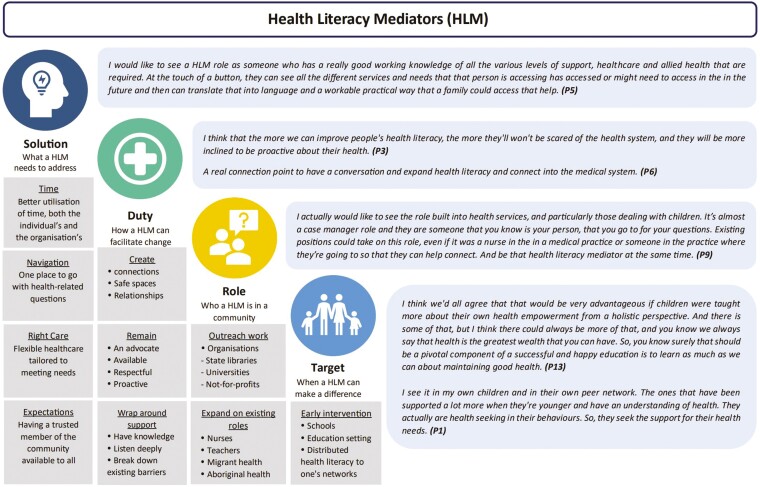
Co-design of the HLM position: stakeholders’ ideas around the expectations and needs for the role.

For the analysis of the data in this qualitative study, a thematic analysis was employed, as described by Braun and Clarke (2019). This method was used to provide insights into how the key stakeholders who participated in the workshops conceptualize an HLM from within their specific contexts. This single thematic analysis involved six distinct phases as outlined by described by Braun and Clarke (2019). Initially, for Phase One, MS and IC (student researcher) collaboratively immersed themselves in the data. This familiarization process included transcribing the workshop discussions verbatim via auto-generation within the Microsoft Teams software and combining that with the researchers’ observational notes and comments that participants had noted in the chat box. It also involved repeatedly listening to audio recordings and thoroughly re-reading the transcripts. Key information from each transcript was highlighted and systematically recorded in an Excel spreadsheet. Subsequently, for Phase Two, IC developed codes. An inductive approach was adopted, beginning with real-world observations, identifying patterns, and formulating theories based on these patterns. The coding process was repeated multiple times, focusing on the data while considering the influence of prior knowledge from earlier readings on the topic. As the analysis progressed and entered Phase Three, IC conducted a theme search, using the codes as foundational elements to the group and refine them into preliminary themes. These initial themes were reviewed and discussed with MS, ensuring that the most pertinent points were captured and aligned with the research objectives as per Phases Four and Five. The final step, Phase Six, involved gathering all qualitative responses, revising the original themes through discussions amongst all authors, and refining and defining the themes to be reported. Through this, one thematic analysis of the workshop discussion’s multiple themes was identified, the themes are reported in Figures 1 and 2, utilizing a contemporary ‘infographic’ structure, to display the findings. Included example quotes were selected for their clarity and precision in reflecting one of the defined themes, although other participants provided similar responses (Braun and Clarke 2019).

## RESULTS

All of the participants could relate to the families presented in each of the cases, with multiple comments identifying how realistic the scenarios were for people living within their community. For example:

*I could identify with this case study (vignette). We all experience our own health issues and those of our family members from time to time. And sometimes it can be very challenging to find the time and know where to go to get the help you need in that moment. So, I think it’s actually quite a common problem for anyone that has to engage with the health system—*Participant 2.

Discussions led to the key stakeholders voicing their thoughts and concerns with the current healthcare system which then allowed us to identify barriers that the individuals and their families in each vignette were facing. Discussion then moved into how the emerging HLM role may assist in overcoming these issues. All stakeholders voiced their opinions on what the expectation of the HLM role should be and how they could see the role helping their own communities.

### Barriers to healthcare

Through analysis of stakeholder discussions, four major themes emerged that encapsulate the barriers to healthcare within the vignettes presented:

#### Theme One: Time

Both individual and systemic challenges in the time to access healthcare exist. On an individual level, limited time due to work, caregiving, and other responsibilities often causes healthcare to be deprioritized. Systemically, long waiting times, limited after-hours services, and delays in accessing specialists or diagnostic tests exacerbate these issues. These barriers are particularly pronounced in rural or regional areas, where healthcare requires additional travel time.

#### Theme Two: Navigating and understanding healthcare

Individuals often face challenges in understanding the available options when seeking healthcare, which can make it difficult to ask the right questions to obtain appropriate care. This issue is exacerbated by limited services throughout healthcare, particularly in regional areas.

#### Theme Three: Access to the right healthcare

It is crucial for individuals to find the right healthcare provider who can offer the necessary information to manage their individual or family’s healthcare needs. Access to care is further altered by financial and social resources.

#### Theme Four: Expectation of healthcare

Individuals expect to be listened to by healthcare professionals. However, when they feel that this is not happening, they are put off and become disengaged with the system. Additionally, societal attitudes can play a role in shaping what is considered socially acceptable regarding illness and chronic disease. This can alter both how healthcare professionals discuss the topics but also how individuals seek help for themselves.

For each of the themes that were identified, there were a number of subthemes that related to the perceived barriers to healthcare. The themes, subthemes, and some example quotes can be seen in Fig. 1.

### Co-design of the HLM position

Building on the identified barriers, discussions then transitioned to how an HLM could play a transformative role in mitigating these challenges and improving overall HL. Stakeholders emphasized four primary expectations for the HLM role:

#### Expectation One: Solution-focused

The HLM role should be solution-focused, using their understanding of individual and community barriers to address healthcare challenges. Stakeholders emphasized that the HLM should empower individuals, families, and communities by providing essential learning opportunities. A key responsibility of the HLM would be improving the efficiency of both individuals’ and healthcare organizations’ time. By streamlining processes and facilitating access to resources, the HLM could help people navigate the complex healthcare system more easily. As a reliable point of contact for health-related inquiries, the HLM would reduce confusion and simplify access to information. Additionally, the HLM could advocate for flexible, community-tailored healthcare solutions. By understanding the specific needs of the population, they could help design strategies that are culturally sensitive and effective. The presence of a trusted community member in this role fosters trust and reliability. Equitable healthcare is central to the HLM’s role, ensuring that all individuals, regardless of background, have equal access to healthcare resources, and are empowered to make informed decisions. By addressing health inequities driven by SDH, the HLM can promote a more inclusive healthcare environment.

#### Expectation Two: Duty to facilitate change

An HLM should play a key role in connecting individuals, families, and communities with healthcare services, bridging gaps between people, healthcare providers, and community organizations. By fostering open communication, the HLM would create safe spaces where individuals feel heard and empowered to ask questions, and making informed health decisions. Their role would involve building trusting relationships and advocating for inclusive, respectful care that addresses the diverse needs of the community. Their role could then extend beyond mere advocacy; they could provide wrap-around support by possessing in-depth knowledge of the healthcare system, actively listening to community members’ problems, strengths, and queries, and working to break down existing barriers to healthcare access.

#### Expectation Three: Community-based role

The key stakeholders wanted to see an HLM as someone in a community who serves as a crucial link between individuals and the healthcare system, enhancing the community’s overall HL. This position could be effectively filled by expanding the responsibilities of existing roles such as nurses, school nurses, teachers, or more specialized positions like migrant health workers and Aboriginal health workers. Building the capacity of these individuals could allow them to step into the role of HLM, utilizing their existing trust and presence within the community. Additionally, larger organizations such as state libraries, universities, and not-for-profits could support these mediators through outreach initiatives, providing resources and training to enhance their effectiveness. The community-based nature of this role would ensure greater success and sustainability, as the HLM could tailor their approaches to the specific needs and cultural contexts of their communities.

#### Expectation Four: Targeted position to have the most impact

The final expectation was that an HLM could play a pivotal role in enhancing HL by intervening early in an individual’s life and be available to individuals during their childhood education years. By providing guidance and education before health needs arise, HLM would help foster a deeper understanding of health-related issues. This proactive approach could be particularly effective in settings such as schools, youth groups, and teenage-specific programmes, where young people are developing independence and forming lifelong habits. By equipping these individuals with the necessary HL skills, they can become informed decision-makers, capable of navigating the healthcare system. Furthermore, the stakeholders identified that as these individuals share their knowledge within their networks, a ripple effect occurs, leading to a more health-literate community overall.

Discussion around how the role could impact the individuals and families presented in the vignettes not only produced four strong expectations above, but also produced other recommendations and supporting ideas for the role. These can be visualized in Fig. 2.

## DISCUSSION

This study aimed to co-design the emerging HLM role for the Tasmanian community and to assess the support, expectations, and need for such a role to then help guide the implementation in the future. This study demonstrates how considering a community’s current HL and engaging key stakeholders (those working in Tasmania’s health sector, with relevant knowledge, experience, and interest in HL and improving the health of their community) in the planning and design of new public health solutions can support the development of a fit-for-purpose, context-specific role. Experiences with similar roles in other parts of the world highlight valuable lessons for the development and sustainability of HLMs. These experiences underscore the importance of incorporating community-specific knowledge and adapting roles to local contexts. In Romania, the Roma Health Mediators initiative serves as a notable case where health mediators have strengthened connections between marginalized communities and health services (Roma Health Mediators Project 2014). This programme has demonstrated that the success of such roles often depends on robust training, community acceptance, and ongoing support. However, these programmes also demonstrate that while community-based health workers can enhance trust and access, they also face challenges related to sustainability, training, and funding. These insights could inform the design and implementation of HLMs in Tasmania, emphasizing the need for a strong framework that considers the social and cultural factors unique to each community.

The HLM role could then go beyond navigation and connection to acknowledge the SDH and could enhance individuals’ HL assets to improve their health outcomes. Addressing SDH through an HLM could play a crucial role in reducing health inequities and improving health outcomes. Social determinants, such as socioeconomic status, education, and living conditions, significantly influence health outcomes (WHO 2024). By focusing on these non-medical factors, HLMs can help bridge the gap between healthcare access and the broader social environment that affects individual and community health. HLMs could play a vital role in creating equitable health opportunities by helping to empower individuals with knowledge and resources to navigate their social contexts effectively. This approach not only addresses immediate health needs but also tackles the root causes of health disparities, promoting long-term health equity (Braveman and Gottlieb 2014, Gnepp et al. 2020). The co-design of the HLM position brings us a step closer to developing an HL-responsive role in Tasmania. This may play a crucial part in improving the HL and health outcomes for the community. The co-designed workshops generated a number of important concepts, from the expectations of the HLM, recommendations for the HLM role, and finally other ideas that would be important for the HLM position’s development.

In order to be successful and sustainable, the role of HLMs must be assessed within Tasmania’s current healthcare landscape. Existing gaps in HL and accessibility indicate where HLMs could be most impactful. HLMs could collaborate with health navigators, social workers, and other healthcare professionals to complement rather than duplicate efforts, enhancing coordination and resource use. However, barriers such as funding, training needs, and acceptance within the community need to be considered and addressed to facilitate effective integration.

### Addressing expectations

An HLM could significantly enhance health outcomes by addressing critical areas such as time management, navigation, right care, and community trust. These issues have been recognized in previous papers and give researchers and policy-makers a starting point when creating practical solutions to address the inequity that exists within health and healthcare (Bhati et al. 2023, Ezeamii et al. 2024, Spencer et al. 2024c). By optimizing time utilization, HLMs may assist both individuals and organizations to focus on essential health-related tasks without unnecessary delays, thereby improving efficiency and productivity. HLMs could serve as a central resource for health-related inquiries, simplifying navigation by providing a single point of contact, which reduces the complexity often associated with accessing healthcare services (Lastrucci et al. 2019). This approach could help to ensure that individuals receive the right care tailored to their specific needs, enhancing the quality of healthcare delivery (Berete et al. 2024). Furthermore, HLMs build community trust by being accessible and reliable members who understand local nuances and concerns (Pasay-an et al. 2024). Setting clear expectations for HLMs is crucial in aligning stakeholders, ensuring that all parties have a shared understanding of the HLM’s role and responsibilities. This alignment fosters collaboration among individuals, communities, and organizations, which could lead to more coordinated efforts and improved health outcomes. By establishing these expectations from the outset, HLMs could effectively bridge gaps in healthcare delivery and empower communities to overcome barriers related to SDH.

HLMs should play a crucial role in creating connections, advocating for individuals, and breaking down barriers within communities. By fostering effective communication, HLMs could help to enable individuals to express themselves and be heard, which is essential for building trust and empowering communities (Bradshaw et al. 2022). Advocacy should be a fundamental aspect of HLMs’ duties, as they work to protect and promote the rights of individuals, ensuring that their voices are amplified in health-related discussions (Pettus and de Lima 2020). This advocacy involves not only supporting individuals in navigating complex health systems but also pushing for systemic changes that address broader health inequities. Breaking down barriers requires HLMs to listen deeply and provide tailored support that respects the unique needs of each community member. Clear communication is vital in achieving these duties, as it ensures that all stakeholders are aligned and informed about the goals and processes involved (Berete et al. 2024). By maintaining open lines of communication, HLMs could effectively coordinate efforts across various sectors, ultimately leading to more inclusive and equitable health outcomes for all community members.

### Role in community

HLMs could significantly expand their roles in various community settings such as state libraries, universities, not-for-profits, and other organizations by integrating with existing roles like nurses, teachers, and health navigators. Libraries serve as accessible hubs for information dissemination, making them ideal partners for HLMs to collaborate with librarians to provide HL resources tailored to a community’s needs (Jenkins et al. 2023). Not-for-profit organizations offer another avenue for HLMs to reach underserved populations by working with health connectors and coaches to deliver targeted interventions. Integrating lessons learned from similar initiatives could help define whether HLMs should be volunteers or professionals and highlight potential challenges. The experiences of health mediators in Eastern Europe, for instance, illustrate that while volunteer-based roles foster community trust, they can suffer from high turnover and inconsistent support (Kósa et al. 2020). On the other hand, structured, professional approaches, like those in multidisciplinary models, provide stability but can be more costly and require significant investment.

In schools and universities, HLMs could work alongside educators to embed HL into curricula, ensuring that students across disciplines develop essential skills for navigating health information (Otten et al. 2024). By utilizing the expertise of nurses and teachers who already play pivotal roles in health education, HLMs could create a more cohesive approach to improving HL (Spencer et al. 2021). This integration could not only enhance the effectiveness of existing programmes but also ensure a comprehensive strategy that addresses the diverse needs of communities, ultimately leading to improved health outcomes and reduced disparities (Ziersch et al. 2020).

### Timing for impact

The HLM role could make a significant impact through early interventions in educational settings and community initiatives. By focusing on schools and educational environments, HLMs could integrate HL into the curriculum, fostering a culture of informed health decision-making from a young age (Otten et al. 2024). This early engagement is crucial as it equips students with the necessary skills to navigate health information throughout their lives, thereby reducing health disparities linked to SDH (Berete et al. 2024). In community settings, HLMs could initiate programs that address specific local health challenges, tailoring strategies to meet the unique needs of diverse populations. Long-term engagement in these communities is essential to build trust and ensure sustained improvements in HL. Tailored strategies that consider cultural, social, and economic factors are necessary for these interventions to be effective (Coughlin et al. 2020). By maintaining an ongoing presence and adapting approaches based on community feedback, HLMs can ensure that their efforts lead to meaningful and lasting changes in health outcomes. This proactive approach not only empowers individuals but also strengthens community resilience against health inequities.

### Recommendations and future research

In summary, these findings suggest that policy-makers could consider incorporating HLMs into policies as a strategic approach to improving public health outcomes. However, the nature of the HLM role—whether as volunteers or professionals—must be critically examined. Integration of the role may follow a spectrum ranging from volunteerism, enhancing the capabilities of current workers without significant cost, and extending to purposely paid professionals who provide dedicated, consistent, and expert support. This spectrum allows for flexible implementation tailored to community needs and resources, balancing cost, sustainability, and impact. While volunteer HLMs could foster trust and connection within communities due to their grassroots nature, research indicates that volunteerism comes with challenges, including limited availability, high turnover, and inconsistent training (Bhattacharya et al. 2022). Upskilling existing professionals to take on HLM responsibilities can enhance workforce capacity, improve continuity of care, and provide cost-effective, immediate HL support within the current system. Employing professional HLMs provides more stability and comprehensive expertise but raises concerns regarding sustainability and costs. Ensuring trust between professionals and community members would also need strategic efforts (Smith and Wallace 2023). Future initiatives could consider a hybrid model where HLMs start as trained volunteers with pathways to professional roles, which would balance trust-building with sustainability. Also, each health or community setting may require an assessment of their current resource requirements, existing skills, and capacity building needs prior to introducing an HLM role. This sort of assessment may support the success and sustainability of such interventions.

For successful implementation, it would be crucial to develop clear guidelines and training programmes that equip HLMs with the necessary skills and resources. These programmes could outline the specific competencies needed, with adaptations for either volunteer or professional tracks, ensuring that all HLMs are prepared to navigate their roles effectively. Drawing on the successes and challenges experienced by health mediator programmes in Eastern Europe, it is evident that best practices should be tailored to local needs. Programmes like the Roma Health Mediators Project emphasize the importance of sustainable training and support structures, which would be critical for the HLM role in Tasmania. Future research should explore the specific challenges of integrating HLMs into various community contexts, including potential resistance from existing healthcare structures and the need for sustainable funding models. It would be valuable to investigate funding strategies that could support either volunteer or paid HLMs, such as community grants, partnerships with local organizations, or government subsidies. Additionally, evaluating the long-term impact of HLM interventions on health outcomes will be essential in refining their role and maximizing their effectiveness in reducing health disparities.

The future of health promotion is poised for transformative impact, emphasizing a holistic approach that integrates education, community engagement, and policy development (Barry 2021). A realistic pathway for the evolution of the HLM role could account for resource constraints and community expectations. Informed by this research the research team will develop a clear position description which will include the roles and responsibilities of an HLM to ensure practical implementation. An HLM could play a pivotal role in this evolution by addressing SDH and empowering individuals with the knowledge and skills needed to navigate complex health systems. Establishing trust and credibility within communities will be crucial, whether the HLMs are volunteer-based or part of a professional workforce. As health promotion strategies continue to evolve, they will increasingly focus on creating supportive environments and strengthening community actions (WHO 2021). By integrating into diverse settings such as schools, workplaces, and community centres, HLMs may support early interventions and tailor strategies to meet the unique needs of different populations. This proactive approach not only addresses immediate health concerns but could also lay the groundwork for sustained improvements in public health outcomes, ultimately contributing to a healthier, more equitable society. Additionally, informed by this research pilot programmes should be considered to assess the feasibility and impact of different HLM approaches, enabling the identification of the most effective structure for Tasmania.

### Strengths and limitations

This study utilizes a co-design approach, which is a significant strength as it ensures that the perspectives of both users and providers are incorporated into the planning and design of the HLM role. By grounding the co-design process in local knowledge and expertise, the study was able to develop context-specific solutions that are more likely to create a HL-responsive environment tailored to the unique needs of the Tasmanian community. This participatory approach promotes stakeholder buy-in and enhances the relevance of the proposed solutions to the local population. However, there are limitations to this approach. While the co-design process generated expectations, recommendations, and ideas based on stakeholders’ personal knowledge and experiences, it does not provide empirical evidence of the effectiveness or feasibility of the HLM role. Consequently, further research is needed to implement and evaluate this role to determine its potential impact on health and equity outcomes within communities. Additionally, the vignettes used in this study were based on only five scales of the HLQ. This approach, while focused, might have excluded other important HL strengths and challenges, potentially affecting the comprehensiveness of the findings and any future decisions informed by this data. Furthermore, the participant group composition was limited, with only one male participant, which could introduce biases. The findings may therefore have limited generalizability, and caution should be exercised when interpreting or applying these results to other contexts. While conducting online workshops via Microsoft Teams facilitated engagement with diverse stakeholders across Tasmania, it may have inadvertently limited participation from those with restricted access to digital technology or low digital literacy. This constraint highlights a potential barrier to inclusive participation. Future research should consider employing a hybrid model that combines in-person and online engagement options to accommodate stakeholders’ preferences, thereby enhancing participation and ensuring a more comprehensive representation of perspectives and capturing different viewpoints.

## CONCLUSION

In conclusion, the HLM role represents a significant opportunity to address health inequities by enhancing time management, streamlining healthcare navigation, ensuring appropriate care delivery, and fostering community trust. By bridging gaps between individuals and healthcare systems, advocating for equity, and tailoring support to community needs, HLMs could play a transformative role in breaking down barriers to healthcare. Their integration into diverse community settings, such as libraries, schools, and universities, alongside collaborations with existing roles like nurses, teachers, and social workers, underscores their potential to amplify HL efforts.

Stakeholders identified key expectations for the HLM role, including its focus on being solution-oriented, community-based, and targeted towards populations with the greatest need. Furthermore, embedding HLMs in educational and early intervention initiatives highlights the importance of long-term engagement and proactive strategies to build a health-literate population. These findings emphasize the need for a structured and sustainable implementation of the HLM role to promote equitable access to health resources and improved public health outcomes. Future research and pilot programmes will be essential to refine this role and evaluate its impact on reducing health disparities.

## Data Availability

The data that support the findings of this study are available on request from the corresponding author. The data are not publicly available due to privacy or ethical restrictions.
